# Discovery of a Novel Multi-Strains Probiotic Formulation with Improved Efficacy toward Intestinal Inflammation

**DOI:** 10.3390/nu12071945

**Published:** 2020-06-30

**Authors:** Michele Biagioli, Adriana Carino, Cristina Di Giorgio, Silvia Marchianò, Martina Bordoni, Rosalinda Roselli, Eleonora Distrutti, Stefano Fiorucci

**Affiliations:** 1 Department of Surgical and Biomedical Sciences, University of Perugia, via L. Severi 1, 06132 Perugia, Italy; michele.biagioli@live.it (M.B.); adriana.carino@unipg.it (A.C.); cristi.digiorgio@gmail.com (C.D.G.); silvia4as@hotmail.it (S.M.); mbordoni92@gmail.com (M.B.); 2 Department of Pharmacy, University of Naples "Federico II", via D. Montesano 49, 80131 Naples, Italy; rosellirosalinda@yahoo.it; 3 Azienda ospedaliera di Perugia, 06132 Perugia, Italy; eleonoradistrutti@katamail.com

**Keywords:** probiotics, inflammatory bowel disease, animal studies, cytokines and inflammation, treg

## Abstract

Dysbiosis is commonly detected in patients with inflammatory bowel disease (IBD), supporting the concept that a dysregulated immune reaction to bacterial antigens has a pathogenic role in the development of intestinal inflammation. In the present study, we have investigated the beneficial effects of a novel probiotic formulation assembled by combining four probiotics (*Streptococcus thermophilus*, *Lactobacillus casei*, *Bifidobacterium breve, Bifidobacterium animalis subsp. Lactis*) with *Bacillus subtilis*, a Gram-positive bacterium, with extensive bio-applications. Mice rendered colitic by administration of TNBS or DSS were administered with *Bacillus subtilis* alone, Vivomixx® or the novel Five strains formulation. Vivomixx® attenuated the severity of inflammation and reduced the development of signs and symptoms of colitis in both models. Adding *Bacillus subtilis* to Vivomixx® improved the beneficial effects of the bacterial therapy. The novel Five strains formulation was as effective as Vivomixx® in reducing the development of signs and symptoms of colitis and reduced the expression of pro-inflammatory mediators including Il-6 and Tnf-α while increased the expression of Il-10 mRNA and the number of Treg. In summary, we have shown that a novel Five strains probiotics formulation exerts beneficial effects on two chemical models of colitis, establishing *Bacillus subtilis* as a probiotic in rodent models of inflammation.

## 1. Introduction

Crohn’s disease and ulcerative colitis are the two main phenotypes of inflammatory bowel disease (IBD), a chronic and relapsing inflammatory disorder of the intestine. Although the precise etiology of IBD is not yet fully understood, the most accepted hypothesis of its pathogenesis is that it develops due to an altered response to antigens deriving from the intestinal microbiota in genetically predisposed individuals [1]. Importantly, alterations in microbiota composition, referred to as dysbiosis, are commonly detected in IBD patients, especially in Crohn’s disease, enforcing the concept that a deregulated immune reaction to bacterial antigens exerts a mechanistic role in the pathogenesis of IBD [2,3,4]. As such, the intestinal microbiota might represent an important therapeutic target in these disorders. 

IBDs are currently treated by immunosuppressive drugs, including local and systemic corticosteroids, anti-inflammatory agents, anti-cytokines therapies and biological agents that target the trafficking of leukocytes towards the gastrointestinal microcirculation [5,6,7]. Despite these efforts, and an impressive pipeline of novel therapeutic approaches, there is still a large subset of patients that are unresponsive or that have incomplete response to treatment or relapse at the end of treatment.

Currently, also the probiotics have entered IBD therapy intending to modulate the composition of the intestinal microbiota and the response of the host’s immune system [8]. Although the mechanisms of action of probiotics are not yet fully understood, many formulations of single strains or combinations of multiple strains are commercially available [9]. The results of clinical trials evaluating the use of probiotics in IBD patients, however, have documented that although probiotics are generally safe, they are only moderately or not effective in treating IBD [8]. Indeed, while beneficial results have been reported in UC (ulcerative colitis)patients and pouchitis, a substantial lack of efficacy has been documented in CD (Crohn’s disease) patients, highlighting the need to identify single strains or combinations of multiple strain probiotic formulations with improved clinical efficacy that will be beneficial and effective in treating the majority of IBD patients [10,11].

About the formulation with a single strain, *Bacillus subtilis* represents one of the most studied strains for the beneficial effects exerted on IBDs. Results from mouse models have provided evidence that *Bacillus subtilis* effectively protects against development of immune dysfunction and signs and symptoms of colitis in rodent models of colitis caused by dextran sodium sulfate (DSS). In this model, feeding with *Bacillus subtilis* promoted an increased biodiversity of the intestinal microbiota, recovery of intestinal tight junctions, along with enhanced generation of beneficial bacterial products such as short chain fatty acids (SCFAs) [12,13,14,15,16]. These beneficial effects were supported by a decreased production of pro-inflammatory cytokines such as Il-6 and Il -17 along with an increased generation of anti-inflammatory mediators such as Il-10 and Tgf-β [12,13,14,15,16], leading to the polarization of intestinal macrophages towards a tolerogenic, anti-inflammatory, M2 phenotype [13]. 

In addition to *Bacilus subtilis*, other probiotic strains such as *Streptococcus thermophilus* [17,18,19,20], *Lactobacillus casei* [21,22,23,24,25], *Bifidobacterium breve* [26,27,28] and *Bifidobacterium animalis* [29,30,31] have proven effective in reducing intestinal inflammation in pre-clinical models of IBDs, suggesting that a combination of *Bacillus subtilis* with these strains, by combining different metabolic and immune-modulatory effects [8], would exert an enhanced anti-inflammatory activity in comparison to the single strain formulation. *Bacillus subtilis* belong to the *Bacillaceae* family who are spore forming bacteria that are supposed to germinate in the living intestine unlike the *Bifidocateria* which are available as lyophilized preparations of vegetative cells [32]. Based on this background, we have worked out a multi-strains formulation containing the Five probiotic strains mentioned above. We tested this new various strain formulation in mouse models of colitis by comparing it with Vivomixx®, a commercially available multi-strains formulation that contains eight probiotic strains with known beneficial effects on colitis [33] used as a positive control, and against a *Bacillus subtilis* single strain formulation.

## 2. Materials and Methods 

### 2.1. Probiotics

Many different formulations of probiotics were used in this study. Vivomixx® was commercially available in pharmacies in Italy and the lot number was 1919101 and expiration date 31/01/2021. For Vivomixx®, the list of strains as they appear on commercial packaging is as follows: *Streptococcus thermophilus* DSM24731®, *Lactobacillus plantarum* DSM24730®, *Bifidobacterium breve* DSM24732®, *Lactobacillus paracasei* DSM24733®, *Lactobacillus delbrueckii subsp. bulgaricus* DSM24734®, *Lactobacillus acidophilus* DSM 24735®, *Bifidobacterium longum* DSM24736®, *Bifidobacterium infantis* DSM24737®. The batch was maintained according to the manufacturer instructions until used. The novel formulation, indicated as “Five strains probiotics”, included the following five strains (percentages): *Streptococcus thermophilus* (30%), *Lactobacillus casei* (30%), *Bifidobacterium breve* (15%), *Bifidobacterium animalis subsp. Lactis* (15%) and *Bacillus subtilis* (10%). All bacteria were produced by Bioprox, France. We have chosen *Bifidocterium animalis subsp. lactis* instead of *Bifidobacterium longum (Bl-O4)* and *infantis* (Bi-07) because the last two species show a 100% identity with *Bifidobacterium animalis subsp*. *lactis* and have been reclassified as such [34,35,36]. 

### 2.2. Animals and Colitis Protocols

Balb/c mice were from Charles River (Italy). The colonies were maintained in the animal facility of the University of Perugia. Mice were housed under controlled temperatures (22 °C) and photoperiods (12:12-hour light/dark cycle), allowed unrestricted access to standard mouse chow and tap water and allowed to acclimate to these conditions for at least 7 days before inclusion in an experiment. The study was conducted in agreement with the Italian law and the protocol was approved by an ethical committee of the University of Perugia and by a National Committee of Italian Ministry of Health permit n° 1126/2016-PR. The health and body conditions of the animals were monitored daily by the veterinarian in the animal facility. Only male mice were used in each experiment. The study protocol caused minor suffering; however, animals losing more than 25% of their initial body weight were euthanized. Colitis was induced in Balb/c mice by administration of 2,4,6-trinitrobenzene sulfonic acid TNBS (Sigma Chemical Co, St Louis, MO, USA), for an acute colitis model, or dextran sulfate, sodium salt DSS (Affymetrix, Cleveland, OH, USA, molecular mass 40–50 kDa) for a chronic colitis model. Animals were monitored daily. For the TNBS colitis model, Balb/c mice were fasted for 12 h (day −1). The day after (day 0), mice were anesthetized, and a 3.5 F catheter inserted into the colon such that the tip was 4 cm proximal to the anus. To induce colitis, 1 mg of TNBS in 50% ethanol was administered via catheter into the lumen using a 1 ml syringe (injection volume of 100 μL); control mice received 50% ethanol alone. At the end of the experiment, the surviving mice were sacrificed, the colon was excised, weighed and evaluated for macroscopic damage. In some groups of mice, probiotics were administered orally at the concentration of 50 × 10^9^ probiotic cfu/kg of body weight dissolved in saline solution [33,37,38,39]. The treatments were administered daily from day 0 to the day of sacrifice. In the DSS-chronic colitis model, DSS was administered in drinking water at 3% for two cycles of 7 days each, interspersed with 7 days of administration of water only. After randomization, mice were administered Vivomixx® or Five strains probiotics by o.s. at the concentration of 50 * 10^9^ probiotic cfu/kg of body weight dissolved in saline solution from day 4 to the end of the experiment. At the end of the experiments, the surviving mice were sacrificed, blood samples were collected by cardiac puncture and the colon was excised, weighed and evaluated for macroscopic damage. In both models, the severity of colitis was measured each day for each mouse by assessing body weight, fecal blood and stool consistency. Each parameter was scored from 0 to 4 as described previously [33,37]. 

### 2.3. Histology

Samples of distal colon (2–3 cm from the anus) were fixed in buffered formalin, cut into 5-µm-thick sections (150 µm between each section, four to eight per fragment per colon), and stained with H&E. The histological score of the colon was assessed as previously described by U. Erben et al [40]. This score evaluated the level of tissue inflammation in relation to the extension of the inflammatory cell infiltrate (mild severity if the cellular infiltrate is only present in the mucosa, moderate if they are involved mucosa and submucosa, marked if the infiltration is transmural), and epithelial changes of the intestinal mucosal architecture like erosions and ulcerations.

### 2.4. Quantification of Fecal Lcn-2 by ELISA

Freshly collected or frozen fecal samples were reconstituted in PBS containing 0.1% Tween 20 (100 mg/mL) and vortexed for 20 min to get a homogenous fecal suspension. These samples were then centrifuged for 10 min at 12,000 rpm and 4 °C. Clear supernatants were collected and stored at −20 °C until analysis. Lipocalin (Lcn)-2 levels were estimated in the supernatants using Duoset murine Lcn-2 ELISA kit according to the manufacturer’s instructions (R&D Systems, Minneapolis, MN) [41].

### 2.5. Isolation of Lamina Propria Cells

The cells were isolated from the colon lamina propria using the Lamina Propria Dissociation Kit (Miltenyi Biotec, Bergisch Gladbach, Germany 130-097-401), according to the instructions.

### 2.6. Flow Cytometry

Flow cytometry analyses were carried out using a three-laser configuration Thermo Fisher Scientific Attune Nxt flow cytometry system. Data were analyzed using the FlowJo software (TreeStar). The gates were set using the fluorescence minus one (FMO) control strategy. FMO controls are samples that include all conjugated Abs present in the test samples except one. The channel in which the conjugated Ab is missing is the one for which the FMO provides a gating control. The following mAbs were used: CD3 PerCP-Cy5.5 (145-2C11, eBioscience, San Diego, CA, USA); CD4 APC-eFluor 789 (GK1.5, eBioscience, San Diego, CA, USA); CD8 Super Bright 702 (53-6.7, Invitrogen, Carlsbad, CA, USA); CD11b FITC (M1/70, BioLegend); Gr1 BV510 (RB6-8C5, BioLegend); CD11c Pe-Cyanine7 (N418, eBioscience, San Diego, CA, USA); CD206 PE (MR6F3, Invitrogen, Carlsbad, CA, USA); FoxP3 APC (FJK-16s, eBioscience, San Diego, CA, USA) and IL-10 Alexa Fluor 700 (JES5-16E3, eBioscience, San Diego, CA, USA). 

### 2.7. Reverse Transcription of mRNA and Real-Time PCR

Colon samples and mesenteric lymph nodes were immediately frozen in liquid nitrogen and stored at −80 °C until used, mechanically homogenated in 1 ml of Trizol (Thermo Scientific™, Waltham, MA, USA). The RNA was extracted according to the manufacturer’s protocol. After purification from genomic DNA by DNase-I treatment (Thermo Scientific™, Waltham, MA, USA), 1 µg of RNA from each sample was reverse-transcribed using random hexamer primers with Superscript-II (Thermo Scientific™, Waltham, MA, USA) in a 20 μL reaction volume; 10 ng cDNA was amplified in a 20 μL solution containing 200 nM of each primer and 10 μL of SYBR Select Master Mix (Thermo Scientific™, Waltham, MA, USA). All reactions were performed in triplicate, and the thermal cycling conditions were as follows: 3 min at 95 °C, followed by 40 cycles of 95 °C for 15 s, 56 °C for 20 s and 72 °C for 30 s, using a Step One Plus machine (Applied Biosystem, Foster City, CA, USA). The relative mRNA expression was calculated according to the 2^(−ΔCt) method comparing the expression of different genes to that of GAPDH housekeeping. Primers were designed using the software PRIMER3 (http://frodo.wi.mit.edu/primer3/) using published data obtained from the NCBI database. The primers used were conducted as follows (forward and reverse): GAPDH (for CTGAGTATGTCGTGGAGTCTAC; rev GTTGGTGGTGCAGGATGCATTG; IFN-γ (for GCTTTGCAGCTCTTCCTCAT; rev ATCCTTTTGCCAGT), TNF-α (for CCACCACGCTCTTCTGTCTA; rev AGGGTCTGGGCCATAGAACT), IL-6 (for CTTCACAAGTCGGAGGCTTA; rev TTCTGCAAGTGCATCATCGT), IL-1β (for GCTGAAAGCTCTCCACCTCA; rev AGGCCACAGGTATTTTGTCG), TGF-β (for TTGCTTCAGCTCCACAGAGA; rev TGGTTGTAGAGGGCAAGGAC), IL-10 (for CCCAGAAATCAAGGAGCATT; rev CTCTTCACCTGCTCCACTGC), FoxP3 (for TCTTCGAGGAGCCAGAAGAG; rev AGCTCCCAGCTTCTCCTTTT).

### 2.8. Metagenomic Analysis

DNA extraction. The microbial DNA was purified from stool samples taken from mice from separate cages, using the PureLink Microbiome DNA Purification Kit (Thermo Scientific™, Waltham, MA, USA), according to the manufacturer’s instructions. Briefly, approximately 100 mg of mouse stool was weighed and transferred to the bead tube and mixed thoroughly with 700 µL of S1-Lysis Buffer and 100 µL of S2-Lysis Enhancer to create a homogeneous sample and incubated at 65 °C for 10 min. The bead tubes were homogenized for 10 min at maximum speed on the horizontal vortex mixer, then centrifuged at 14,000× *g* for 5 min and 400 µL of supernatant was transferred to a clean micro-centrifuge tube and vortexed immediately with 250 µL of S3-Cleanup Buffer. After 2 min of centrifugation, 500 µL of supernatant was transferred in a new Eppendorf and mixed with 900 µL of S4-Binding Buffer. Then, 700 µL of sample mixture was loaded onto a spin column tube and centrifuged at 14,000× *g* for 1 min (2X). The spin column was then washed with 500 µL of S5-Wash Buffer and the flow-through was discarded. Finally, the spin-column was placed in a clean tube, and the purified DNA was eluted with 100 µL of S6-Elution Buffer. The isolated DNA was quantified with a Qubit dsDNA HS Assay Kit on Qubit 3.0 Fluorometer (Thermo Scientific™, Waltham, MA, USA) according to the manufacturer’s instructions and then stored at −20 °C. 

16S rRNA sequencing. Sequencing was performed using Ion 16S Metagenomics Kit (Thermo Scientific™, Waltham, MA, USA) on the Ion Torrent S5 platform (Thermo Scientific™, Waltham, MA, USA). Briefly, 3ng of DNA was subjected to amplification of 16S rRNA libraries using two primer pools to amplify seven hypervariable regions of bacterial 16S rRNA. Primers were partially digested and barcoded adapters (Ion Xpress Barcode Adapters 1-16 Kit) ligated to the amplicons, using the Ion Plus Fragment Library Kit (Thermo Scientific™, Waltham, MA, USA), purified using the Agencourt AMPure XP beads (Beckman Coulter, Brea CA, USA) according to the manufacturer’s protocol, and stored at −20 °C until further processing. The concentration of each 16S library was determined by qPCR using the Ion Library Quantitation Kit and a Qubit 3.0 fluorometer (Thermo Scientific™, Waltham, MA, USA). The library was diluted to ~100 pM before template preparation. Template preparation of the barcoded libraries was performed using the Ion Chef and the Ion S5 System (Thermo Scientific™, Waltham, MA, USA). A maximum of 16 barcoded 16S samples were sequenced on an Ion 520 chip (Thermo Scientific™, Waltham, MA, USA) using the Ion 510 & Ion 520 & Ion 530 Kit-Chef (Thermo Scientific™, Waltham, MA, USA) according to the manufacturer’s instructions. 

Metagenomics analysis. Automated analysis, annotation and taxonomical assignments were generated using Ion Reporter Software-Metagenomics Workflow (Ion Reporter 5.10.2.0). The Ion Reporter Software enables the rapid identification (at genus or species level) of microbes present each sample, using both curated Greengenes and premium curated MicroSEQ ID 16S rRNA reference databases. The Ion Reporter metagenomics workflow also provides primer information, classification information, percent ID and mapping information. 

Data visualization and statistical analyses of taxonomy. Data visualization and statistical analyses were performed using Krona and QIIME™ analysis software, and related packages were used for diversity and correlation analyses. Principal coordinates analysis (PCoA) was conducted with identified reads/OTUs using classical multidimensional scaling (Bray–Curtis) to analyze the distribution of dissimilarities and analysis of variance using abundance data. 

### 2.9. Statistical Analysis

An ANOVA followed by a non-parametric Mann–Whitney U test or a two-tailed unpaired Student t test were used for statistical comparisons (* *p* < 0.05) using the Prism 6.0 software (GraphPad, La Jolla, CA, USA).

## 3. Results

### 3.1. Effects of the Probiotics Formulations in Mouse Model of Acute Colitis

We have first investigated the efficacy of the Five strains probiotics formulation in an acute model of colitis, caused by intrarectal administration of TNBS, a widely used model of Th1-mediated disease with some similarities with Crohn’s disease. To compare the efficacy of the novel formulation with existing probiotics, groups of 5–7 mice were also dosed daily with *Bacillus substilis* alone or Vivomixx® alone or in combination with *Bacillus subtilis*. 

**Figure 1:**
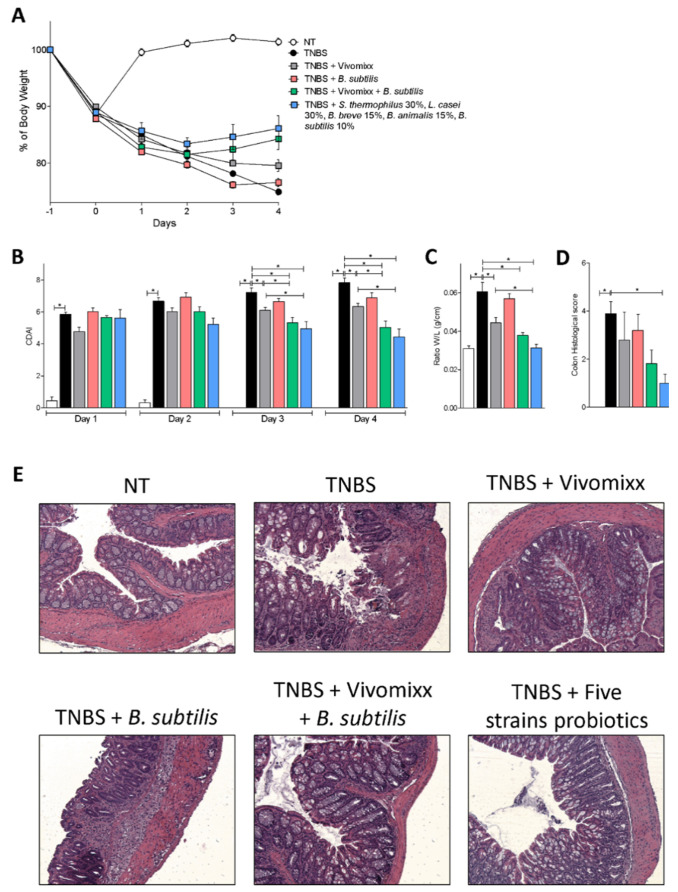
Effects of probiotics blends on acute colitis. Acute colitis was induced by administering TNBS on BALB/c mice. Co-treatment with different formulations of probiotics by gavage from day 0 to day 4. Changes in body weight (**A**), colitis disease activity index (CDAI) (**B**) of mice during the course of TNBS-induced colitis, ratio of colon weight/colon length (**C**) and colon histological score (**D**). Results are the mean ± SEM of 5–7 mice per group. * *p* < 0.05. H&E staining of colon sections from control mice, mice treated with TNBS, and mice treated with TNBS plus different probiotics (original magnification 10×) (**E**).

The severity of the TNBS-induced colitis was assessed daily by monitoring changes in body weight and colitis disease activity index (CDAI) and colonic macroscopic and microscopic features at the time of sacrifice. The data shown in Figure 1 highlight the protective effect of Vivomixx®, as already shown in previous studies [33]. However, the best beneficial effect was obtained by dosing the mice with the Five strains probiotics, which effectively attenuated the body weight loss by ≈15% and the CDAI by 45% at day 4 compared with the group treated with TNBS alone (Figure 1A,B). Furthermore, the Five strains probiotics improved the macroscopic and microscopic features of the colitis (Figure 1C–E). The combination of Vivomixx® with *Bacillus subtilis*, one of the components of the Five strains probiotics mixture, heighted the beneficial effects exerted by Vivomixx® alone, without matching the effects exerted by the Five strains probiotics (Figure 1). Conversely, the administration of a formulation consisting of *Bacillus subtilis* alone exerted a minor beneficial effect (Figure 1).

**Figure 2:**
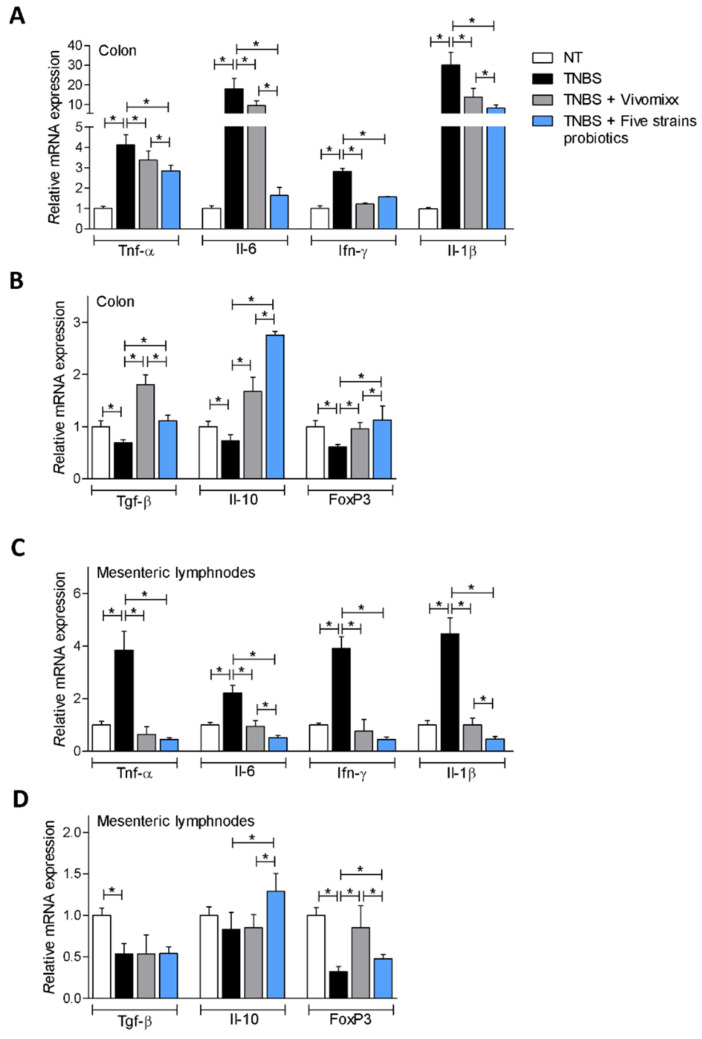
Impact on the gene expression of Vivomixx® and Five strains probiotics on acute colitis. In BALB/c mice acute colitis was induced by administering TNBS. Mice were co-treated with Vivomixx® and the Five strains probiotics by gavage from day 0 to day 4. Relative mRNA expression of pro-inflammatory genes Tnf-α, Il-6, Ifn-γ, and Il-1β and anti-inflammatory genes Tgf-β, Il-10 and FoxP3 in colon (**A**,**B**) and mesenteric lymph nodes (**C**,**D**) was assayed by real-time PCR. Data are normalized to Gapdh mRNA. Results are the mean ± SEM of 5–7 mice per group. * *p* < 0.05.

These data were confirmed by the analysis of the expression of pro- (Tnf-α, Il-6, Ifn-γ and Il-1 β) and anti-inflammatory (Tgf-β, Il-10 and FoxP3) biomarkers in the colon and mesenteric lymph nodes (mLN) of mice rendered colitic by TNBS and treated with Vivomixx® or with the Five strains probiotics (Figure 2). The results of these assays demonstrated that while exposure to TNBS alone increased the expression of pro-inflammatory mediators Il-6 (≈20 folds) and Il-1β (≈30 folds) (Figure 2A), this pattern was reversed by both probiotic formulations. Furthermore, the Five strains probiotics was significantly more effective than Vivomixx® in reducing the level of Il-6 (Figure 2A). Both probiotic formulations increased the expression of anti-inflammatory genes Tgf-β, Il-10 and FoxP3 (Figure 2B). Furthermore, both probiotic formulations reduced the expression levels of the pro-inflammatory cytokines and increased the expression of FoxP3 in the mesenteric lymph nodes. In contrast, none of the treatments had any effect on Tgf-β expression, but the novel formulation increased Il-10 gene expression (Figure 2C,D).

### 3.2. Effects of the Probiotic Formulations in a Model of Chronic Colitis

**Figure 3:**
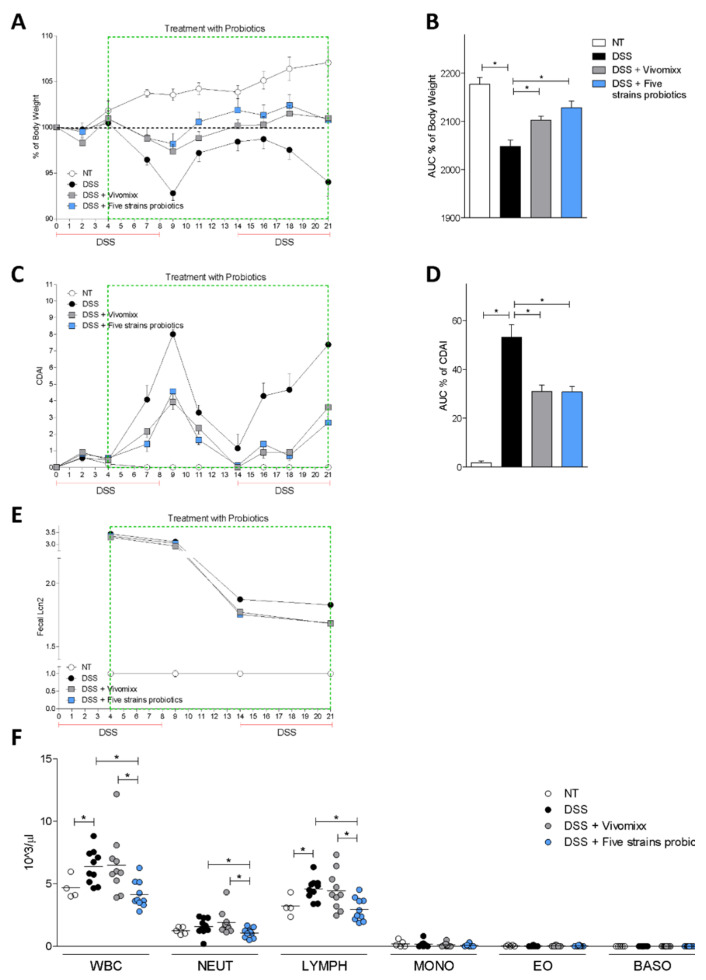
Effects of probiotics on chronic colitis. The experiment was carried out on BALB/c mice. Mice were treated with DSS in drinking water for two cycles of 7 days each and then administered with vehicle or one of the different formulations of probiotics by gavage from day 4 to the end of the experiment. Data shown: changes in body weight (**A**), area under the curve (AUC) of % of body weight (**B**), CDAI (**C**) and area under the curve of CDAI (**D**) during the course of DSS-induced colitis. Lipocalin 2 levels in stool at different time points (**E**). Blood cells counted at the end of experiments (day 21) (**F**). Results are the mean ± SEM of 7-11 mice per group. * *p* < 0.05.

To further investigate the efficacy of the two formulation in another model of colitis, we have dosed Vivomixx® and the Five strains probiotic mixture to mice administered DSS in the drinking water for three weeks. The results of this second experiment demonstrated that the signs and symptoms of colitis assessed by measuring body weight and CDAI, and the levels of Lcn2, a biomarker of colon inflammation (Figure 3A–E), were equally attenuated by Vivomixx® and by the Five strains probiotics mixture. Additionally, while DSS increased the total white blood cells (WBC) and lymphocytes, the Five strains probiotics, but not Vivomixx®, prevented these changes (Figure 3F).

**Figure 4:**
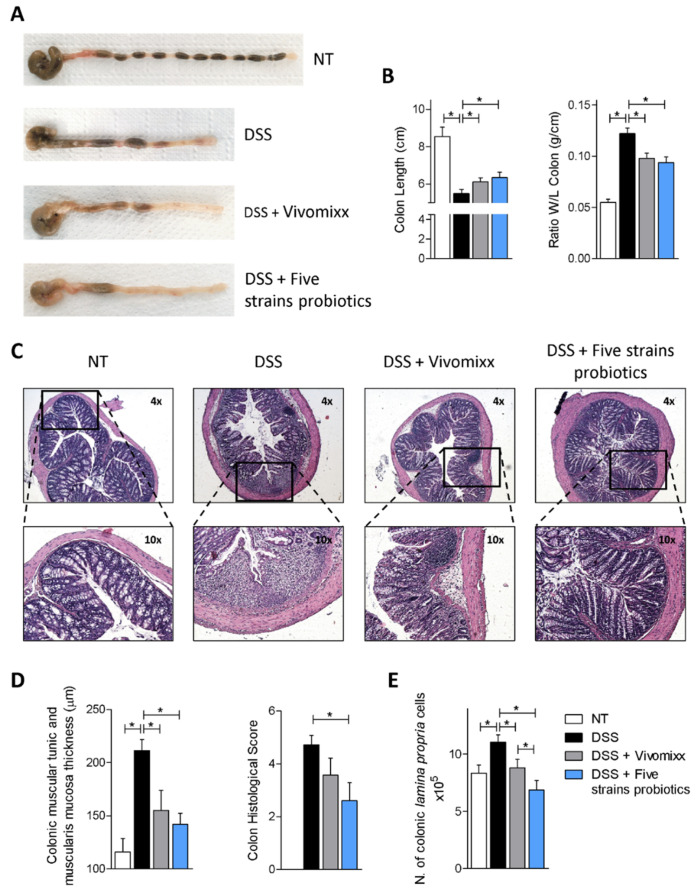
Effects of Vivomixx® and the Five strains probiotics on macroscopic and microscopic characteristics of the colon. BALB/c mice were treated with DSS in drinking water for two cycles of 7 days each and then administered with vehicle or one of two probiotics formulations by gavage from day 4 to the end of the experiment. Photographs of colon from control, DSS-treated and DSS plus Vivomixx® and Five strains probiotics-treated mice (**A**), colon length and ratio of colon weight/colon length (**B**). H&E staining of colon sections from each experimental group (original magnification ×4 and ×10) (**C**). Evaluation of wall thickness and histological score (**D**), and number of *lamina propria* cells (**E**). Results are the mean ± SEM of 7–11 mice per group. * *p* < 0.05.

The analysis of the length and weight of the colon confirmed the beneficial effect of the probiotic administration (Figure 4A,B). Histopathology analysis of the colon sections, obtained from different experimental groups, confirmed a strong leukocyte infiltration and loss of normal architecture of the colonic wall in DSS-treated mice (Figure 4E). Both Vivomixx® and the Five strains probiotics reversed these features as well as macroscopic features (Figure 4C,D). Furthermore, both formulations effectively reduced leukocyte trafficking toward the colon as measured by assessing the number of the *lamina propria* cells (Figure 4E). All these data on the colon macroscopic and microscopic features showed that the Five strains probiotics exerted a greater beneficial effect compared with Vivomixx® (Figure 4).

**Figure 5:**
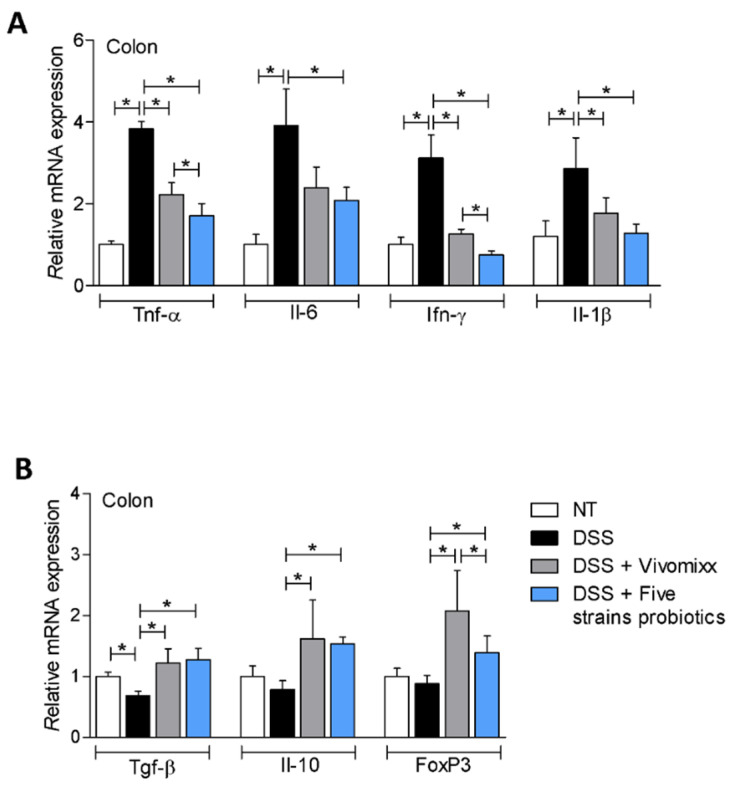
Probiotics modulate the expression of pro and anti-inflammatory genes in the colon. BALB/c mice were treated with DSS in drinking water for two cycles of 7 days each and then administered with vehicle or probiotics formulations by gavage from day 4 to the end of the experiment. Relative mRNA expression of pro- and anti-inflammatory genes was assessed by real-time PCR in the colon: Tnf-α, Il-6, Ifn-γ, Il-1β (**A**) and Tgf-β, Il-10, FoxP3 (**B**). Results are the mean ± SEM of 7–11 mice per group. * *p* < 0.05.

To gain further insights on the mechanisms that support the beneficial effects of probiotic formulations in this model, we have then investigated the expression of pro- and anti-inflammatory genes in the colon and composition of the colon *lamina propria* cells by flow cytometry (Figure 5). Again, while exposure to DSS increased the expression of Tnf-α, Il-6, Ifn-γ and Il-1β (Figure 5A), this pattern was reduced by treatment with both probiotic formulations, although the Five strains probiotics exhibited a greater anti-inflammatory activity compared with Vivomixx®, as indicated by enhanced modulation of Tnf- α and Ifn-γ (Figure 5A). On the other hand, both probiotics increased the expression of Tgf- β, Il-10 and FoxP3 (Figure 5B), though Vivomixx® was highly effective in boosting FoxP3 expression (Figure 5B). 

**Figure 6:**
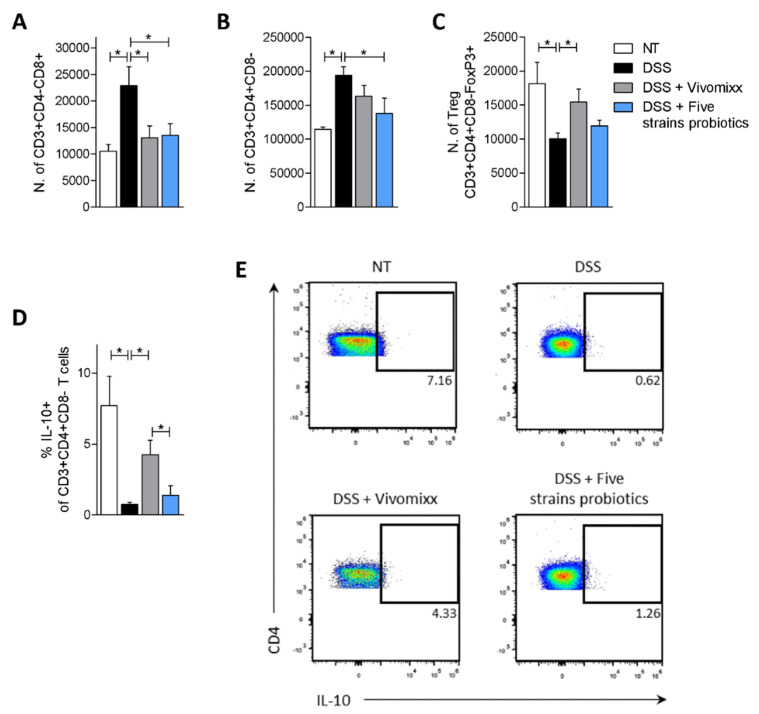
Probiotics modulate the phenotype of T lymphocytes in *lamina propria*. BALB/c mice were treated with DSS in drinking water for two cycles of 7 days each and then administered with vehicle or probiotics formulations by gavage from day 4 to the end of the experiment. Colon samples were used to perform a detailed characterization of cells composition of colonic *lamina propria* by IC-FACS analysis. Data shown are numbers of CD3^+^CD4^-^CD8^+^ T cells (**A**), CD3^+^CD4^+^CD8^-^ T helper cells (**B**), Treg cells (CD3^+^CD4^+^CD8^-^FoxP3^+^) (**C**) and percentage of IL-10^+^ T helper cells (**D**). Flow cytometry analysis of CD4 and IL-10 expression in CD3^+^ T cells (**E**) derived from colonic *lamina propria*. Results are the mean ± SEM of 7–11 mice per group. * *p* < 0.05.

**Figure 7:**
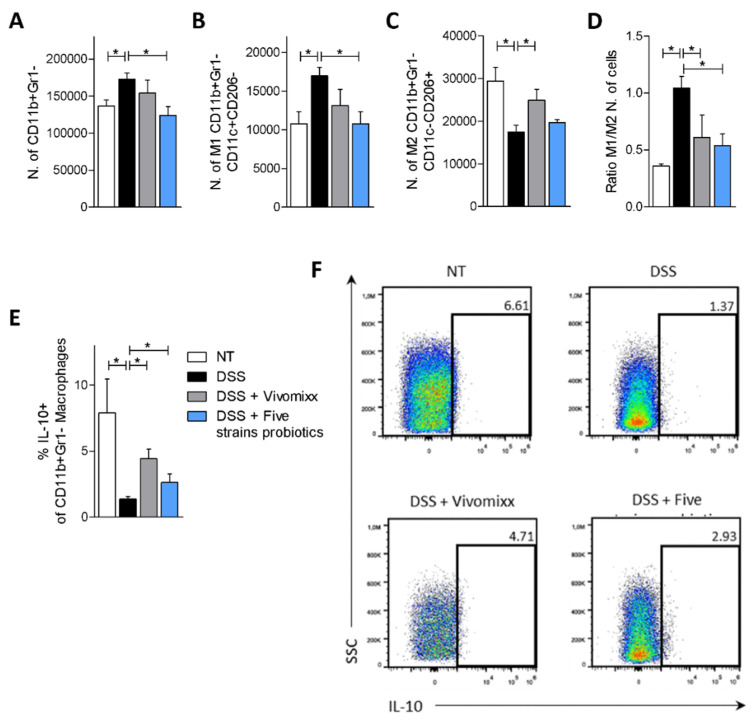
Probiotics modulate the phenotype of macrophages in *lamina propria*. BALB/c mice were treated with DSS in drinking water for two cycles of 7 days each and then administered with vehicle or probiotics formulations by gavage from day 4 to the end of the experiment. Colon samples were used to perform a detailed characterization of cells composition of colonic *lamina propria* by IC-FACS analysis. Number of total macrophages CD11b^+^Gr1^-^ (**A**), M1 subpopulation (CD11b^+^Gr1^-^CD11c^+^CD206^-^) (**B**), M2 macrophages (CD11b^+^Gr1^-^CD11c^-^CD206^+^) (**C**), ratio between M1/M2 number of macrophages (**D**) and percentage of IL-10^+^ macrophages (**E**). Flow cytometry analysis of IL-10 expression in CD11b^+^Gr1^-^ macrophagic cells (**F**). Results are the mean ± SEM of 7–11 mice per group. * *p* < 0.05.

Furthermore, a detailed flow cytometry analysis of *lamina propria*-infiltrating cells (Figure 6, Figure 7 and Figure S1) demonstrated that both probiotic formulations effectively reduced the number of CD8^+^ T cells (CD3^+^CD4^-^CD8^+^) and CD4^+^ T helper cells (CD3^+^CD4^+^CD8^-^) (Figure 6A,B), but had no significant effect on the percentage of these cells (Figure S1A,B). Next, we have also investigated the effects of the two formulations on modulating T helper cell subsets. The results of this analysis demonstrated that only Vivomixx® increased the number of *lamina propria* (CD3^+^CD4^+^CD8^-^FoxP3^+^) Treg cells (Figure 6C) and the percentage of IL-10^+^ T helper cells (CD3^+^CD4^+^CD8^-^IL-10^+^) (Figure 6D,E). 

We have then investigated the effect of *in vivo* treatment with probiotics on macrophages, the largest population of immune cells most found in the *lamina propria* of the colon [42,43] (Figure 7). Of interest, both formulations of probiotics reduced the number of total macrophages (CD11b+Gr1-) and the number of M1 subsets (CD11b^+^Gr1^-^CD11c^+^CD206^-^) detected in the colon of mice administered DSS (Figure 7A,B). However, only the Five strains probiotics had a statistically significant effect (Figure 7A,B). In contrast, we were unable to detect change either on the percentage of total marginalized macrophages in the *lamina propria* or on the percentage of the M1 subpopulation (Figure S1D). On the other hand, both probiotic mixtures increased the number and the frequency of M2 macrophages (CD11b^+^Gr1^-^CD11c^-^CD206^+^) and the percentage of IL-10^+^ macrophages as demonstrated by the decrease in the ratio of M1/M2 macrophages (Figure 7C–F and Figure S1D). Together, these data suggest that Vivomixx® exerted a greater effect in inducing the expansion of anti-inflammatory macrophages if compared with the Five strains probiotics (Figure 7C–F). 

**Figure 8:**
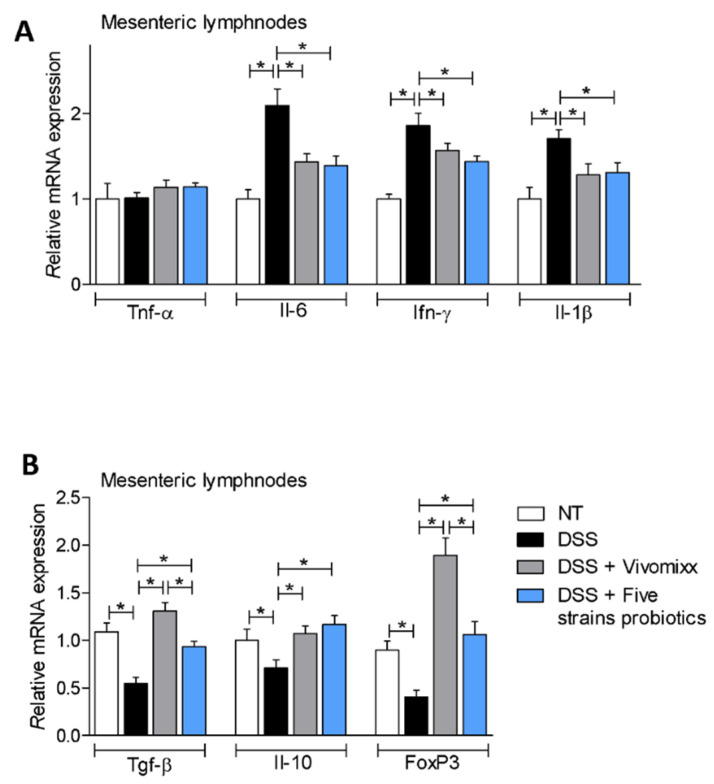
Impact of DSS-induced colitis and probiotic formulations in the peripheral immune system. The experiment was carried out on BALB/c mice. Mice were treated with DSS in drinking water for two cycles of 7 days each and then administered with vehicle or probiotics by gavage from day 4 to the end of the experiment. Quantitative real-time PCR analysis of the expression of Tnf-α, Il-6, IFN-g and Il-1β (**A**) and Tgf-β, Il-10, and Foxp3 (**B**) genes in mLN. The data are normalized to Gapdh mRNA. Results are the mean ± SEM of 7–11 mice per group. * *p* < 0.05.

Analysis of the expression of pro- and anti-inflammatory genes in the mesenteric lymph nodes were consistent with these findings (Figure 8A,B), as the two probiotic formulations reversed the pro-inflammatory pattern caused by treating mice with DSS. Additionally, both probiotics increased the expression of anti-inflammatory genes Tgf- β, Il-10 and FoxP3 (Figure 8A,B). 

### 3.3. Impact of the two Probiotic Formulations on the Composition of the Intestinal Microbiota

**Figure 9:**
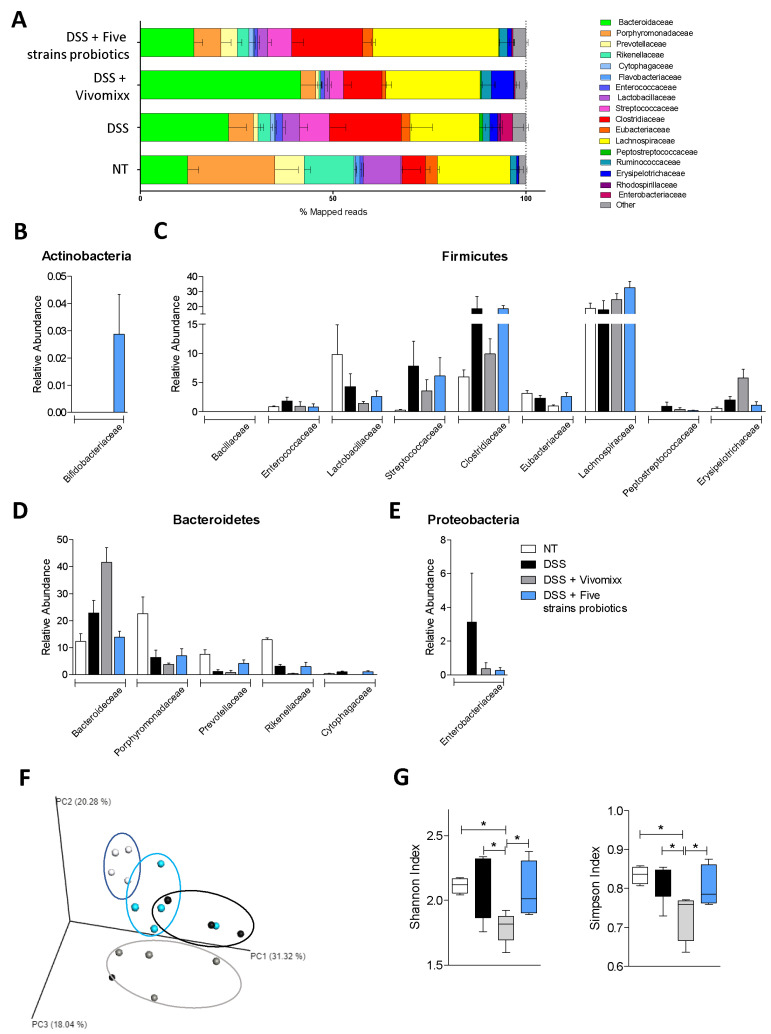
Effects of probiotic formulations on microbiome composition. The experiment was carried out on BALB/c mice. Mice were treated with DSS in drinking water for two cycles of 7 days each and then administered with vehicle or probiotics by gavage from day 4 to the end of the experiment. Stool samples were collected at the end of the experiment (day 21). The gut microbiota composition profiles at family level expressed as percentage of mapped reads (**A**) and detailed graphs of the most represented families grouped by phylum (**B**–**E**). Plot of principal coordinate analysis (PCoA) of fecal microbiota using the Bray–Curtis dissimilarity matrices at family levels (**F**). Shannon and Simpson indices used to estimate the α-diversity of fecal microbiota (**G**). Results are the mean ± SEM of 5 mice per group. * *p* < 0.05.

To ascertain whether the beneficial effects exerted by the two probiotic mixtures in the chronic colitis models were mediated by changes in the composition of intestinal microbiota, we have carried out a metagenomic analysis of the composition of the intestinal microbiota from Five mice per group (Figure 9), in fecal samples collected at the end of the study (21 days from start of colitis and 17 days from start of treatment with probiotic formulations). The results of these studies revealed a robust difference in microbiome composition between the various experimental groups expressed as relative abundance of family, calculated as percent of mapped reads (Figure 9A). As displayed in Figure 9, the DSS treatment radically changed the microbiome composition by increasing the relative percentage of *Bacteroidaceae* and reducing the percentage of *Porphyromonadaceae*. Both probiotic formulations modulated the microbiome composition compared with DSS-treated mice, but exerted different effects on the composition as shown in the Figure 9B–E. The PCoA plot of β diversity analysis at the family level (Bray–Curtis analysis) showed a minor dissimilarity between untreated mice and mice treated with the Five strains probiotics, whereas Vivomixx® administration was significantly less effective in reversing the effect of DSS on family composition (Figure 9F). Furthermore, the analysis of the alpha diversity using the Shannon and Simpson indices showed that while DSS did not change the biodiversity and richness of fecal microbiota, Vivomixx® significantly decreased the alpha diversity of the microbial community, whereas the Five strains probiotics reshaped the biodiversity and the stability of microbiota by increasing both the Shannon and Simpson indices.

## 4. Discussion

IBD are chronic intestinal inflammatory disorders characterized by dysregulated immune responses to the intestinal microbiota that occurs in genetically susceptible hosts. Dysbiosis, a quantitative and qualitative alteration of the intestinal microbiota structure, is recognized as a causative factor in IBD [44] (.). Consistent with this view, several studies have shown that most of IBD patients have increased levels of potentially pathogenic *Proteobacteria* (including *Enterobacteriaceae* such as *E. coli* and *Klebsiella*), *Fusobacteria* and pathogenic fungi [45], along with reduced levels of putative beneficial Firmicutes such as *Faecalibacterium prausnitzii* and *Clostridium clusters IV and XIVa*, among others [46,47,48]. Together with the demonstration that a microbiota colonization is required to develop colitis in germ-free susceptible rodents [4,49], these data provide a strong rationale for therapeutically modifying the enteric microbiota in IBD patients [8,50]. Several therapeutic strategies based on the use of single strain or multiple strains probiotics, prebiotics and symbiotics along with fecal microbiota transplantation have been adopted in IBD patients [51,52,53]. Furthermore, novel live biotherapeutics, among which several putative beneficial bacteria including *Clostridium, Firmicutes spores, Bacteroides* and *Roseburia* isolated from healthy human microbiota are currently investigated [54]. Analyses of randomized clinical trials with various probiotic formulations have demonstrated that single strain probiotics exert beneficial effects over placebos in UC patients [55] and that beneficial effects were maintained also when the probiotics were associated with anti-inflammatory agents such as 5-aminosalicycilic acid [56]. Multiple strains formulations have been demonstrated effective in UC and pouchitis. Indeed, VSL#3, a blend of eight strains, *L. casei*, *L. plantarum*, *L. acidophilus*, *L. delbrueckii* subspecies *bulgaricus*, *B. longum*, *B. breve*, *B. infantis*, and *S. thermophilus*, has been shown effective in improving remission and reducing relapse rates in UC and patients with pouchitis [33,37]. However, recent studies have shown that due to changes in the production site and bacterial composition, VSL#3 is no longer effective in reducing intestinal inflammation in preclinical models of IBD [33,37]. Therefore, in the present study, we have used Vivomixx®, a commercially available multi-strains probiotic preparation made with the original formulation (known as De Simone’s formulation) used in the original VSL#3 [33,57]. 

Chemical c*olitides* induced in Balb/c mice by TNBS or DSS mimic several “clinical” and histopathological features of IBD, and have been extensively used to dissect the immune response to intestinal microbiota in preclinical models [58]. Present results demonstrated that administering colitic mice with Vivomixx® attenuated the development of colitis in both the TNBS and DSS models. Indeed, treatment with Vivomixx® alleviated the development of “clinical” signs and symptoms of colitis, reduced inflammation (as measured by assessing the macroscopic and histopathological scores) and induced a regulatory immune response, as highlighted by the increased expression of signature cytokines such IL-10 along with Foxp3, a marker of Treg polarization. The analysis of the expression FoxP3+ CD4 cells in the colon and in the mesenteric lymph nodes, along with the characterization of the cells infiltrating the colon *lamina propria* by flow cytometry (Figure 2, Figure 5, Figure 6 and Figure 8), demonstrated that exposure to Vivomixx® expands the Treg pool as we have demonstrated previously [33]. Adding *Bacillus subtilis* to Vivomixx® improved the beneficial effects of the bacterial therapy in the TNBS model of colitis. The *Bacillus subtilis*–Vivomixx® mixture had additive effects, in comparison with *Bacillus subtilis* alone and Vivomixx® alone, in reducing the body weight lost and the severity of colitis, highlighting the potential beneficial effects of *Bacillus subtilis* in the context of a multi-strains probiotic preparation [12,13,14,15,16].

However, Vivomixx® is a complex blend of eight different bacteria that is produced through a rather sophisticated industrial process whose steps need to be carefully controlled to avoid impact on bacterial metabolism as previously reported [33,37]. A reduction in formulation complexity, and industrial costs, is therefore a desirable step that will be needed to expand the availability of the highly effective and high doses probiotics to IBD patients. For these reasons, we have decided to develop a novel and simplified bacterial formulation consisting of Five different strains: *Streptococcus thermophilus* (30%), *Lactobacillus casei* (30%), *Bifidobacterium breve* (15%), *Bifidobacterium animalis* (15%) and *Bacillus subtilis* (10%). All the strains chosen for the new formulation have been studied extensively and there are a number of studies supporting their beneficial effects in preclinical and clinical settings [17,18,19,20,21,22,23,24,25,26,27] and four of them, excluding *Bacillus subtilis,* are included in the De Simone formulation, and their effectiveness in reducing intestinal inflammation has been validated through a number of studies in preclinical and clinical settings in the last two decades. 

*Bacillus subtilis* is a Gram-positive bacterium that has several attractive properties with high potential in bio-applications and has been recently isolated from the normal human intestinal microbiota [59,60]. *Bacillus subtilis* is a sister species to *Bacillus amyloliquefaciens* (i.e., two species originated by differentiation from a shared progenitor) belonging to the largest group of bacterial species characterized by a high genetic correlation, the so called *Bacillus subtilis* group [61]. The high genetic similarity existing between *Bacillus amyloliquefaciens* and *Bacillus subtilis* species made it difficult to effectively distinguish these two taxonomic groups by comparing the coding sequences for the 16S rRNA [62], which represents the election marker for the taxonomic identification of bacteria [63]. One property of *Bacillus subtilis* is the ability to form architecturally complex communities termed biofilms, which self-produce an extracellular matrix comprised of lipids, proteins exhibiting amyloid-like properties, extracellular DNA and exopolysaccharides [64]. Interestingly, *Bacillus subtilis* can develop biofilms in the gut of living organisms which may help to reset the composition of intestinal microbiota and to protect the intestinal mucosa from aggressive bacteria and reshape intestinal immunity [59,60,65,66,67]. Since the properties of *Bacillus subtilis* were rather unique among the probiotic species, this might explain the enhanced efficacy of various blends obtained by combining canonical probiotics with the *Bacillus subtilis*. The Five strains formulation reported in this study was as effective as Vivomixx® (Figure 1) in reducing intestinal inflammation and inflammation-driven immune dysfunction in the TNBS model of colitis. In contrast to Vivomixx®, that seems to work mostly by expanding the counter-regulatory branch of innate and adaptive immunity, i.e., by promoting the differentiation toward an M2 phenotype of macrophages and a regulatory phenotype of T cells, the novel formulation, not only directly attenuated the inflammatory response as demonstrated by a robust reduction in the expression of Il-6 (Figure 2A,C), but also increased the expression of Il-10 both in the colon and in the mesenteric lymph nodes (Figure 2B,D). This view is further confirmed by the finding that treating DSS mice with the novel formulation not only attenuated the expression of pro-inflammatory mediators but reduced the number of colon *lamina propria* infiltrating cells (Figure 4E) along with the total number of circulating WBC and lymphocytes. Taken together, these data strongly suggest that the novel formulation exerts both anti-inflammatory and counter-regulatory effects which might be compatible with enhanced mucosal protection. Finally, because the novel formulation exerts similar effects of Vivomixx® on the intestinal microbiota structure, we speculated that enhanced beneficial effects could be related to the ability of *Bacillus subtilis* to generate intestinal biofilms which might compete for biofilm formation by pathogenic bacteria. This hypothesis needs to be tested by specifically designed clinical investigations. 

Another limitation of this study is that there are relatively few studies over the safety of *Bacillus subtilis* as a probiotic in human settings. Despite the fact that a closely related *Bacillaceae* member, *Bacillus clausii,* is the main ingredient of several pro-biotic formulations and has been used for treating acute diarrhea [32], the safety and efficacy of *Bacillus subtilis* in IBD patients, needs to be formally assessed and investigated in clinical trials. 

## 5. Conclusions

In conclusion, here we report on the efficacy of a multi-strains formulation based on the use of established probiotics in combination with *Bacillus subtilis*, establishing this bacterium as a probiotic in rodent models of colitis, therefore human clinical trials are needed to determine the benefits on IBD patients. The novel formulation is made by Five bacteria, and presents several advantages over the “classical” eight strains formulation known as the De Simone formulation that has been marketed in the last two decades under several commercial brands. Clinical studies are needed to prove the efficacy and safety in human settings.

## Supplementary Materials

The following are available online at https://www.mdpi.com/2072-6643/12/7/1945/s1, Figure S1: BALB/cmiceweretreatedwithDSSindrinkingwaterfortwocyclesof7dayseachandthenadministeredwithvehicleorprobioticsformulationsbygavagefromday4totheendoftheexperiment.Colon.

## Abbreviation

TNBS	2,4,6-Trinitrobenzenesulfonic acid
DSS	dextran sodium sulfate
H&E	Hematoxilin and Eosin
CDAI	colitis disease activity index
Treg	Regulatory T lymphocytes
mLN	mesenteric lymph nodes

## References

[1] De Souza H.S., Fiocchi C. (2016). Immunopathogenesis of IBD: Current state of the art. Nat. Rev. Gastroenterol. Hepatol..

[2] Frank D.N., St Amand A.L., Feldman R.A., Boedeker E.C., Harpaz N., Pace N.R. (2007). Molecular-phylogenetic characterization of microbial community imbalances in human inflammatory bowel diseases. Proc. Natl. Acad. Sci. USA.

[3] Walker A.W., Sanderson J.D., Churcher C., Parkes G.C., Hudspith B.N., Rayment N., Brostoff J., Parkhill J., Dougan G., Petrovska L. (2011). High-throughput clone library analysis of the mucosa-associated microbiota reveals dysbiosis and differences between inflamed and non-inflamed regions of the intestine in inflammatory bowel disease. BMC Microbiol..

[4] Sartor R.B., Wu G.D. (2017). Roles for Intestinal Bacteria, Viruses, and Fungi in Pathogenesis of Inflammatory Bowel Diseases and Therapeutic Approaches. Gastroenterology.

[5] Danese S., Panés J. (2014). Development of drugs to target interactions between leukocytes and endothelial cells and treatment algorithms for inflammatory bowel diseases. Gastroenterology.

[6] Mosli M.H., Rivera-Nieves J., Feagan B.G. (2014). T-cell trafficking and anti-adhesion strategies in inflammatory bowel disease: Current and future prospects. Drugs.

[7] Fiorucci S., Biagioli M., Distrutti E. (2018). Immunephenotype Predicts Response to Vedolizumab: Integrating Clinical and Biochemical Biomarkers in the Treatment of Inflammatory Bowel Diseases. Dig. Dis. Sci..

[8] Oka A., Sartor R.B. (2020). Microbial-Based and Microbial-Targeted Therapies for Inflammatory Bowel Diseases. Dig. Dis. Sci..

[9] Korada S.K., Yarla N.S., Mishra V., Daim M.A., Sharma B., Gm A., R R., M P., Peluso I., Kamal M.A. (2018). Single Probiotic versus Multiple Probiotics—A Debate On Current Scenario for Alleviating Health Benefits. Curr. Pharm. Des..

[10] Rondanelli M., Faliva M.A., Perna S., Giacosa A., Peroni G., Castellazzi A.M. (2017). Using probiotics in clinical practice: Where are we now? A review of existing meta-analyses. Gut Microbes.

[11] Naseer M., Poola S., Ali S., Samiullah S., Tahan V. (2020). Prebiotics and Probiotics in Inflammatory Bowel Disease (IBD): Where Are We Now And Where Are We Going? Funders. Curr. Clin. Pharmacol..

[12] Zhang H.L., Li W.S., Xu D.N., Zheng W.W., Liu Y., Chen J., Qiu Z.B., Dorfman R.G., Zhang J., Liu J. (2016). Mucosa-reparing and microbiota-balancing therapeutic effect of. Exp. Ther. Med..

[13] Cao G., Wang K., Li Z., Tao F., Xu Y., Lan J., Chen G., Yang C. (2018). Ameliorates Dextran Sulfate Sodium-Induced Colitis by Improving Gut Microbial Dysbiosis in Mice Model. Front. Microbiol..

[14] Wu C., Ouyang M., Guo Q., Jia J., Liu R., Jiang Y., Wu M., Shen S. (2019). Changes in the intestinal microecology induced by bacillus subtilis inhibit the occurrence of ulcerative colitis and associated cancers: A study on the mechanisms. Am. J. Cancer Res..

[15] Jing Y., Liu H., Xu W., Yang Q. (2017). Amelioration of the DSS-induced colitis in mice by pretreatment with 4,4′-diaponeurosporene-producing. Exp. Ther. Med..

[16] Gong Y., Li H., Li Y. (2016). Effects of Bacillus subtilis on Epithelial Tight Junctions of Mice with Inflammatory Bowel Disease. J. Interf. Cytokine Res..

[17] Bailey J.R., Vince V., Williams N.A., Cogan T.A. (2017). Streptococcus thermophilus NCIMB 41856 ameliorates signs of colitis in an animal model of inflammatory bowel disease. Benef. Microbes.

[18] Wasilewska E., Zlotkowska D., Wroblewska B. (2019). Yogurt starter cultures of Streptococcus thermophilus and Lactobacillus bulgaricus ameliorate symptoms and modulate the immune response in a mouse model of dextran sulfate sodium-induced colitis. J. Dairy Sci..

[19] Chen Y., Zhang M., Ren F. (2019). A Role of Exopolysaccharide Produced by. Molecules.

[20] Ito M., Ohishi K., Yoshida Y., Okumura T., Sato T., Yokoi W., Sawada H. (2008). Preventive effect of Streptococcus thermophilus YIT 2001 on dextran sulfate sodium-induced colitis in mice. Biosci. Biotechnol. Biochem..

[21] Thakur B.K., Saha P., Banik G., Saha D.R., Grover S., Batish V.K., Das S. (2016). Live and heat-killed probiotic Lactobacillus casei Lbs2 protects from experimental colitis through Toll-like receptor 2-dependent induction of T-regulatory response. Int. Immunopharmacol..

[22] Bellavia M., Rappa F., Lo Bello M., Brecchia G., Tomasello G., Leone A., Spatola G., Uzzo M.L., Bonaventura G., David S. (2014). Lactobacillus casei and bifidobacterium lactis supplementation reduces tissue damage of intestinal mucosa and liver after 2,4,6-trinitrobenzenesulfonic acid treatment in mice. J. Biol. Regul. Homeost. Agents.

[23] Zhang Y., Hou Q., Ma C., Zhao J., Xu H., Li W., Wang Y., Ma H., Zhang H., Sun Z. (2019). Lactobacillus casei protects dextran sodium sulfate- or rapamycin-induced colonic inflammation in the mouse. Eur. J. Nutr..

[24] Jacouton E., Chain F., Sokol H., Langella P., Bermúdez-Humarán L.G. (2017). Probiotic Strain. Front. Immunol..

[25] Cortes-Perez N.G., Lozano-Ojalvo D., Maiga M.A., Hazebrouck S., Adel-Patient K. (2017). Intragastric administration of Lactobacillus casei BL23 induces regulatory FoxP3+RORγt+ T cells subset in mice. Benef. Microbes.

[26] Zheng B., Van Bergenhenegouwen J., Overbeek S., Van de Kant H.J., Garssen J., Folkerts G., Vos P., Morgan M.E., Kraneveld A.D. (2014). Bifidobacterium breve attenuates murine dextran sodium sulfate-induced colitis and increases regulatory T cell responses. PLoS ONE.

[27] Jeon S.G., Kayama H., Ueda Y., Takahashi T., Asahara T., Tsuji H., Tsuji N.M., Kiyono H., Ma J.S., Kusu T. (2012). Probiotic Bifidobacterium breve induces IL-10-producing Tr1 cells in the colon. PLoS Pathog..

[28] Heuvelin E., Lebreton C., Grangette C., Pot B., Cerf-Bensussan N., Heyman M. (2009). Mechanisms involved in alleviation of intestinal inflammation by bifidobacterium breve soluble factors. PLoS ONE.

[29] Kang J., Chung W.H., Lim T.J., Lim S., Nam Y.D. (2017). Complete genome sequence of the. New Microbes New Infect..

[30] Chae J.M., Heo W., Cho H.T., Lee D.H., Kim J.H., Rhee M.S., Park T.S., Kim Y.K., Lee J.H., Kim Y.J. (2019). Erratum to: Orally-Administered. J. Microbiol. Biotechnol..

[31] Paveljšek D., Juvan P., Košir R., Rozman D., Hacin B., Ivičak-Kocjan K., Rogelj I. (2018). Lactobacillus fermentum L930BB and Bifidobacterium animalis subsp. animalis IM386 initiate signalling pathways involved in intestinal epithelial barrier protection. Benef. Microbes.

[32] Khatri I., Sharma G., Subramanian S. (2019). Composite genome sequence of Bacillus clausii, a probiotic commercially available as Enterogermina. BMC Microbiol..

[33] Biagioli M., Capobianco D., Carino A., Marchianò S., Fiorucci C., Ricci P., Distrutti E., Fiorucci S. (2019). Divergent Effectiveness of Multispecies Probiotic Preparations on Intestinal Microbiota Structure Depends on Metabolic Properties. Nutrients.

[34] Masco L., Ventura M., Zink R., Huys G., Swings J. (2004). Polyphasic taxonomic analysis of Bifidobacterium animalis and Bifidobacterium lactis reveals relatedness at the subspecies level: Reclassification of Bifidobacterium animalis as Bifidobacterium animalis subsp. animalis subsp. nov. and Bifidobacterium lactis as Bifidobacterium animalis subsp. lactis subsp. nov. Int. J. Syst. Evol. Microbiol..

[35] Turroni F., Foroni E., Pizzetti P., Giubellini V., Ribbera A., Merusi P., Cagnasso P., Bizzarri B., De’Angelis G.L., Shanahan F. (2009). Exploring the diversity of the bifidobacterial population in the human intestinal tract. Appl. Environ. Microbiol..

[36] Loquasto J.R., Barrangou R., Dudley E.G., Stahl B., Chen C., Roberts R.F. (2013). Bifidobacterium animalis subsp. lactis ATCC 27673 is a genomically unique strain within its conserved subspecies. Appl. Environ. Microbiol..

[37] Biagioli M., Laghi L., Carino A., Cipriani S., Distrutti E., Marchianò S., Parolin C., Scarpelli P., Vitali B., Fiorucci S. (2017). Metabolic Variability of a Multispecies Probiotic Preparation Impacts on the Anti-inflammatory Activity. Front. Pharmacol..

[38] Talero E., Bolivar S., Ávila-Román J., Alcaide A., Fiorucci S., Motilva V. (2015). Inhibition of chronic ulcerative colitis-associated adenocarcinoma development in mice by VSL#3. Inflamm. Bowel Dis..

[39] Mencarelli A., Distrutti E., Renga B., D’Amore C., Cipriani S., Palladino G., Donini A., Ricci P., Fiorucci S. (2011). Probiotics modulate intestinal expression of nuclear receptor and provide counter-regulatory signals to inflammation-driven adipose tissue activation. PLoS ONE.

[40] Erben U., Loddenkemper C., Doerfel K., Spieckermann S., Haller D., Heimesaat M.M., Zeitz M., Siegmund B., Kühl A.A. (2014). A guide to histomorphological evaluation of intestinal inflammation in mouse models. Int. J. Clin. Exp. Pathol..

[41] Chassaing B., Srinivasan G., Delgado M.A., Young A.N., Gewirtz A.T., Vijay-Kumar M. (2012). Fecal lipocalin 2, a sensitive and broadly dynamic non-invasive biomarker for intestinal inflammation. PLoS ONE.

[42] Bain C.C., Mowat A.M. (2014). The monocyte-macrophage axis in the intestine. Cell. Immunol..

[43] Cerovic V., Bain C.C., Mowat A.M., Milling S.W. (2014). Intestinal macrophages and dendritic cells: What’s the difference?. Trends Immunol..

[44] Manichanh C., Rigottier-Gois L., Bonnaud E., Gloux K., Pelletier E., Frangeul L., Nalin R., Jarrin C., Chardon P., Marteau P. (2006). Reduced diversity of faecal microbiota in Crohn’s disease revealed by a metagenomic approach. Gut.

[45] Hoarau G., Mukherjee P.K., Gower-Rousseau C., Hager C., Chandra J., Retuerto M.A., Neut C., Vermeire S., Clemente J., Colombel J.F. (2016). Bacteriome and Mycobiome Interactions Underscore Microbial Dysbiosis in Familial Crohn’s Disease. mBio.

[46] Lloyd-Price J., Arze C., Ananthakrishnan A.N., Schirmer M., Avila-Pacheco J., Poon T.W., Andrews E., Ajami N.J., Bonham K.S., Brislawn C.J. (2019). Multi-omics of the gut microbial ecosystem in inflammatory bowel diseases. Nature.

[47] Ohkusa T., Okayasu I., Ogihara T., Morita K., Ogawa M., Sato N. (2003). Induction of experimental ulcerative colitis by Fusobacterium varium isolated from colonic mucosa of patients with ulcerative colitis. Gut.

[48] Atarashi K., Tanoue T., Shima T., Imaoka A., Kuwahara T., Momose Y., Cheng G., Yamasaki S., Saito T., Ohba Y. (2011). Induction of colonic regulatory T cells by indigenous Clostridium species. Science.

[49] Kim S.C., Tonkonogy S.L., Albright C.A., Tsang J., Balish E.J., Braun J., Huycke M.M., Sartor R.B. (2005). Variable phenotypes of enterocolitis in interleukin 10-deficient mice monoassociated with two different commensal bacteria. Gastroenterology.

[50] Britton G.J., Contijoch E.J., Mogno I., Vennaro O.H., Llewellyn S.R., Ng R., Li Z., Mortha A., Merad M., Das A. (2019). Microbiotas from Humans with Inflammatory Bowel Disease Alter the Balance of Gut Th17 and RORγt. Immunity.

[51] Laurell A., Sjöberg K. (2017). Prebiotics and synbiotics in ulcerative colitis. Scand. J. Gastroenterol..

[52] Imdad A., Nicholson M.R., Tanner-Smith E.E., Zackular J.P., Gomez-Duarte O.G., Beaulieu D.B., Acra S. (2018). Fecal transplantation for treatment of inflammatory bowel disease. Cochrane Database Syst. Rev..

[53] Moayyedi P., Surette M.G., Kim P.T., Libertucci J., Wolfe M., Onischi C., Armstrong D., Marshall J.K., Kassam Z., Reinisch W. (2015). Fecal Microbiota Transplantation Induces Remission in Patients With Active Ulcerative Colitis in a Randomized Controlled Trial. Gastroenterology.

[54] Cohen L.J., Cho J.H., Gevers D., Chu H. (2019). Genetic Factors and the Intestinal Microbiome Guide Development of Microbe-Based Therapies for Inflammatory Bowel Diseases. Gastroenterology.

[55] Ganji-Arjenaki M., Rafieian-Kopaei M. (2018). Probiotics are a good choice in remission of inflammatory bowel diseases: A meta analysis and systematic review. J. Cell. Physiol..

[56] Peng L., Zhong Y., Wang A., Jiang Z. (2019). Probiotics combined with aminosalicylic acid affiliates remission of ulcerative colitis: A meta-analysis of randomized controlled trial. Biosci. Rep..

[57] Distrutti E., Cipriani S., Mencarelli A., Renga B., Fiorucci S. (2013). Probiotics VSL#3 protect against development of visceral pain in murine model of irritable bowel syndrome. PLoS ONE.

[58] Wirtz S., Popp V., Kindermann M., Gerlach K., Weigmann B., Fichtner-Feigl S., Neurath M.F. (2017). Chemically induced mouse models of acute and chronic intestinal inflammation. Nat. Protoc..

[59] Vlamakis H., Chai Y., Beauregard P., Losick R., Kolter R. (2013). Sticking together: Building a biofilm the Bacillus subtilis way. Nat. Rev. Microbiol..

[60] Hong H.A., Khaneja R., Tam N.M., Cazzato A., Tan S., Urdaci M., Brisson A., Gasbarrini A., Barnes I., Cutting S.M. (2009). Bacillus subtilis isolated from the human gastrointestinal tract. Res. Microbiol..

[61] Fritze D. (2004). Taxonomy of the genus bacillus and related genera: The aerobic endospore-forming bacteria. Phytopathology.

[62] Wang L.T., Lee F.L., Tai C.J., Kuo H.P. (2008). Bacillus velezensis is a later heterotypic synonym of Bacillus amyloliquefaciens. Int. J. Syst. Evol. Microbiol..

[63] Woese C.R. (1987). Bacterial evolution. Microbiol. Rev..

[64] Tam N.K., Uyen N.Q., Hong H.A., Duc l.H., Hoa T.T., Serra C.R., Henriques A.O., Cutting S.M. (2006). The intestinal life cycle of Bacillus subtilis and close relatives. J. Bacteriol..

[65] Shan M., Gentile M., Yeiser J.R., Walland A.C., Bornstein V.U., Chen K., He B., Cassis L., Bigas A., Cols M. (2013). Mucus enhances gut homeostasis and oral tolerance by delivering immunoregulatory signals. Science.

[66] Permpoonpattana P., Hong H.A., Phetcharaburanin J., Huang J.M., Cook J., Fairweather N.F., Cutting S.M. (2011). Immunization with Bacillus spores expressing toxin A peptide repeats protects against infection with Clostridium difficile strains producing toxins A and B. Infect. Immun..

[67] Stasiłojć M., Hinc K., Peszyńska-Sularz G., Obuchowski M., Iwanicki A. (2015). Recombinant Bacillus subtilis Spores Elicit Th1/Th17-Polarized Immune Response in a Murine Model of Helicobacter pylori Vaccination. Mol. Biotechnol..

