# Supplementary material for: Discovery of a Novel Multi-Strains Probiotic Formulation with Improved Efficacy toward Intestinal Inflammation
Source: Nutrients. 2020 Jun 30;12(7):1945. doi: 10.3390/nu12071945 (PMC7400193; doi:10.3390/nu12071945)
Supplement: Supplementary file 1 [file nutrients-12-01945-s001.pdf]

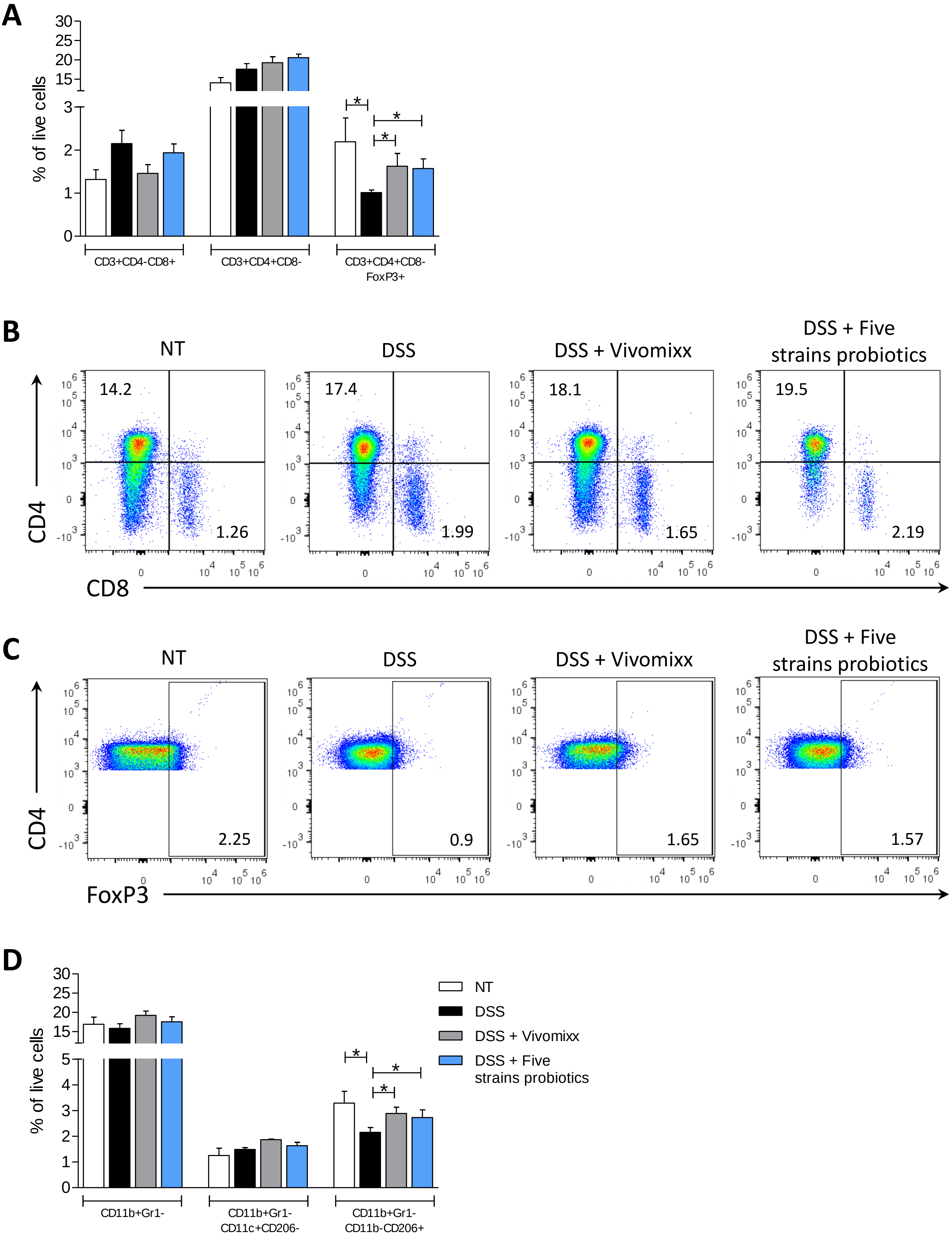

A
% of live cells
CD3+CD4-CD8+
CD3+CD4+CD8-
CD3+CD4+CD8- FoxP3+
B
NT
DSS
DSS + Vivomixx
DSS + Five strains probiotics
14.2
1.26
17.4
1.99
18.1
1.65
19.5
2.19
CD4
CD8
C
NT
DSS
DSS + Vivomixx
DSS + Five strains probiotics
2.25
0.9
1.65
1.57
CD4
FoxP3
D
% of live cells
CD11b+Gr1-
CD11b+Gr1- CD11c+CD206-
CD11b+Gr1- CD11b-CD206+
NT
DSS
DSS + Vivomixx
DSS + Five strains probiotics

Figure S1.

Figure S1. BALB/c mice were treated with DSS in drinking water for two cycles of 7 days each and then administered with vehicle or probiotics formulations by gavage from day 4 to the end of the experiment. Colon samples were used to perform a detailed characterization of cells composition of colonic *lamina propria* by IC-FACS analysis. Data shown are frequency of $CD3^+CD4^-CD8^+$ T cells, $CD3^+CD4^+CD8^-$ T helper cells, Treg cells ($CD3^+CD4^+CD8^-FoxP3^+$) (**A**) and flow cytometry analysis of CD4 and CD8 expression in $CD3^+$ T cells (**B**), and FoxP3 expression in $CD3^+CD4^+$CD8- T helper cells (**C**) derived from colonic *lamina propria*. Frequency of total macrophages $CD11b^+Gr1^-$, M1 ($CD11b^+Gr1^-CD11c^+CD206^-$) and M2 ($CD11b^+Gr1^-CD11c^-CD206^+$) macrophages subpopulation derived from colonic *lamina propria* (**D**). Results are the mean ± SEM of 7-11 mice per group. $^*p < 0.05$.
